# Use of modern magnetic resonance imaging technology for lumbar screw planning

**DOI:** 10.1016/j.bas.2025.105874

**Published:** 2025-11-19

**Authors:** Yorck Rommelspacher, Andrew Dixon, André Pascal Schulte, Stephan Tanner, Frank Schellhammer, Sabine Kling, Peter Seevinck, Marta Gironés Sangüesa, Andreas Christian Strauss

**Affiliations:** a Krankenhaus der Augustinerinnen, Department of Spine Surgery, Jakobstraße 27-31, 50678, Cologne, Germany; b Krankenhaus der Augustinerinnen, Department of Radiology, Jakobstraße 27-31, 50678, Cologne, Germany; c Brainlab AG, Olof-Palme-Straße 9, 81829, Munich, Germany; d UMC Utrecht, Heidelberglaan 100, 3584 CX, Utrecht, Netherlands; e University of Bonn, Department of Orthopaedics and Trauma Surgery, Venusberg Campus 1, 53127, Bonn, Germany; f MRIguidance BV, Maliesingel 23, 3581 BG, Utrecht, Netherlands

**Keywords:** Magnetic resonance imaging, Synthetic computed tomography, Lumbar spine, Screw planning

## Abstract

**Introduction:**

Preoperative screw planning enables the use of modern technologies such as navigation and robotics. To reduce radiation exposure to patients, there is growing interest in Magnetic Resonance Imaging (MRI) technologies.

**Research question:**

This study assesses the use of isotropic MRI and synthetic Computed Tomographies (sCT) for planning lumbar screws.

**Methods:**

Two 3D T1-weighted scans were performed on 22 patients, one isotropic fast spin-echo sequence, and one multi-echo gradient echo sequence for generating sCTs. A total of 200 screws were planned equally split across the isotropic MRIs and sCTs. All scans were then fused to an intraoperative scan for evaluation. Each screw was evaluated by three surgeons using Gertzbein-Robbins classification and a qualitative survey.

**Results:**

A mean interrater agreement of 94.5 % (83 %–100 %) was observed. A significant difference was identified in the Gertzbein-Robbins classification (P = 0.04) where sCT had the most A and B rated screws. The qualitative survey identified differences in screw length and screw positioning but not in screw diameter.

**Discussion and conclusion:**

Nearly 75 % of cases can use modern MRI sequences for planning of lumbar screws. Where the MRI sequence alone is insufficient for total confidence, sCT can be used to supplement the scan and enable effective planning in approximately 90 % of patients without the need for ionizing radiation.

## Highlights

• Modern MRI & sCT enable lumbar screw planning without ionizing radiation.
• Study: 22 patients, 200 screws planned, high interrater agreement (94.5 %).
• SCT showed better screw positioning; diameter & length similar in both.
• MRI alone sufficient in ∼75 %, MRI + sCT in ∼90 % of cases.

## 1 Introduction

Placement of pedicle screws to fixate the spine is an established and often used treatment for developmental, traumatic, and degenerative spinal conditions and has been shown to be safe and effective (Bonello et al., 2023; Modi et al., 2008). Suitable preoperative planning, including assessment of osseous structures, in posterior spinal fusion procedures can identify the optimal screw trajectory, which can improve screw accuracy, enable novel technologies (such as navigation), and simplify operating room preparation (Daemi et al., 2015; Floyd et al., 2020; Patel et al., 2021). Specifically for pedicle screws, the planning process requires review of key bone landmarks and identification of entry and target points for screw placement. As a result, the planning is often performed on CT data, which is able to provide excellent visualization of osseous structures and can have better accuracy for vertebra measurements than traditional MRI (Omar et al., 2020).

Although CT can enable effective screw planning for spinal surgery, it exposes the patient to ionizing radiation (Hall and Brenner, 2008). MRI does not expose the patient to ionizing radiation and is often used for diagnostic purposes as it can delineate soft tissue and important nervous structures (Dinkla et al., 2018; Harada et al., 2020). Although visualization of soft tissues can be beneficial for preoperative planning, the reduced contrast of osseous tissue in MRI can limit screw planning. Often a combination of both CT and MRI is used by surgeons to have a more holistic view of the anatomy and pathology of a patient (Harada et al., 2020; Shanechi et al., 2019; Rajasekaran et al., 2017). In using both CT and MRI preoperatively, there is an increase in the cost, patient burden, and overall clinical workflow time. As a result, clinical workflows which reduce the need for a full suite of imaging are of interest. Improvements to the resolution of MRI have continued over time (Harada et al., 2020; Soh et al., 2021) and it may be possible to use modern sequences to plan screws safely and effectively. To reduce the steps and time of the clinical workflow, new MRI techniques are under investigation, specifically, high resolution MRI sequences and generation of synthetic CTs (sCT) from MRI. Specifically, a novel AI developed by MRIguidance is able to generate a synthetic CT from MRI (Rommelspacher et al., 2025; Davidar et al., 2023; Staartjes et al., 2021; Morbée et al., 2021). Use of sCT provides improved visualization of osseous structures compared to MRI alone, and therefore could potentially be used to improve screw planning as well.

To complete the clinical workflow, once planning has been performed using such MRI technologies, the plan can be fused to an intraoperative CT or Digital Volume Tomography (DVT) scan to enable accurate surgery (van et al., 2015; Schmidt et al., 2021). The aim of this study was to assess whether these modern preoperative MRI techniques can be used for lumbar pedicle screw planning when fused with an intraoperative CT.

## 2 Methods

### 2.1 Overview

**Fig. 1:**
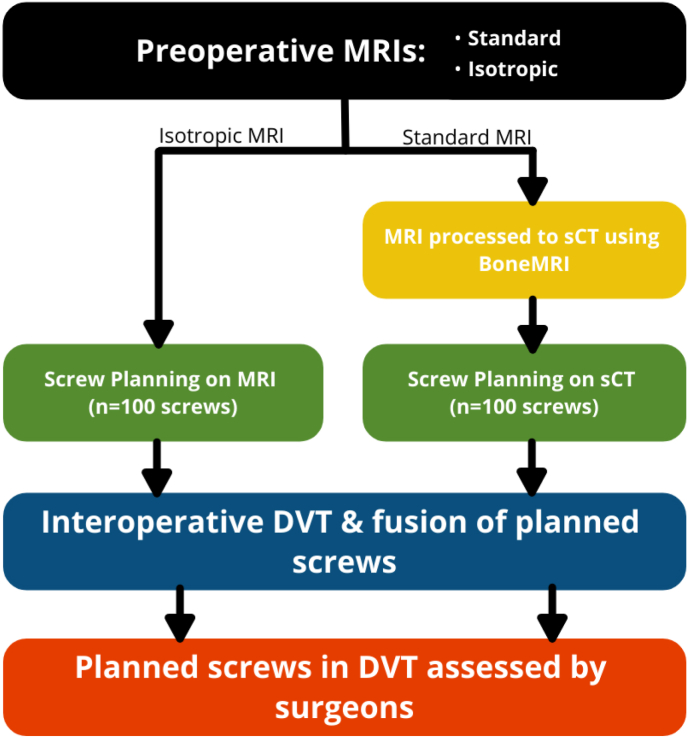
Flowchart of the overall method.

To evaluate screw planning using preoperative MRI technologies, 22 patients were enrolled in the study. A preoperative MRI exam was performed as part of standard of care. The exam included a 3D radiofrequency-spoiled T1-weighted multiple gradient-echo (T1w-MGE) sequence and an isotropic 3D T1w fast spin-echo MRI sequence. The T1w-MGE MRI was processed using the BoneMRI software to generate an sCT (BoneMRI V1.4, MRIguidance BV, Utrecht, NL). Pedicle screws were then planned for each patient on both scan technologies, the isotropic MRI scans and the sCTs. A DVT scan was performed for each patient, which was then fused to each MRI modality of that patient, the isotropic MRI sequence and the sCT. The image fusion shows the planned screws from each technology in the DVT. The placement of screws in the fused image data was reviewed and qualitatively assessed by three expert spinal surgeons. A summary of the overall study methodology is shown in Fig. 1. This study was approved by Ethics Committee of ÄrztekammerNordrhein (nr 201943) and performed in accordance with the declaration of Helsinki. Furthermore, an informed consent form was obtained from each participant.

### 2.2 Patient selection

This was a single center, retrospective analysis from a prospective study, which included a total of 22 patients who were to be treated for degenerative spinal disease at a single institute (Augustinian Hospital, Cologne, Germany). Patients were included if posterior stabilization was planned to be performed using a screw and rod system in the sacrolumbar region of the spine and they were over the age of 18. All patients signed written informed consent.

### 2.3 Image acquisition

Two MRI sequences were performed in this study, first a standard spine MRI examination, with a modified scan protocol to include a T1w-MGE sequence, and an acquisition duration of 4m18s. This T1w-MGE sequence was used for sCT reconstruction using the BoneMRI software. The second MRI scan was an isotropic 3D T1w Fast Spin-Echo MR sequence, with an acquisition duration of 4m36s, replacing a conventional T1w fast spin-echo scan. All MRI scans were acquired with a Magnetom Aera 1.5T MRI system (Siemens Healthineers AG, Erlangen, Germany).

Intraoperative DVT images were performed using Cios spin (Siemens Healthineers AG, Erlangen, Germany) with 400 images in 30 s. The patient was in the supine position.

### 2.4 Screw planning

Screw planning was performed by a single surgeon (YR) with 12 years of experience in spinal surgery. The planning was completed using Spine Planning 1.0 software (Brainlab, Munich-Germany) for each patient on the isotropic MRI scan and the sCT. In the 22 patients included in the study, 100 screws were planned for each scan technology (MRI only and sCT) at corresponding vertebra levels for a patient. To aid assessment of the screws, it was noted if the screw plan used bi-cortical, in-out-in, straightforward, and anatomic trajectories (Perna et al., 2016). Vertebra level and laterality were also recorded for each screw.

After the screw planning was performed, the MRI and sCT datasets were fused by vertebra with the intraoperative DVT using an Image Fusion software (Brainlab, Munich-Germany).

**Fig. 2:**
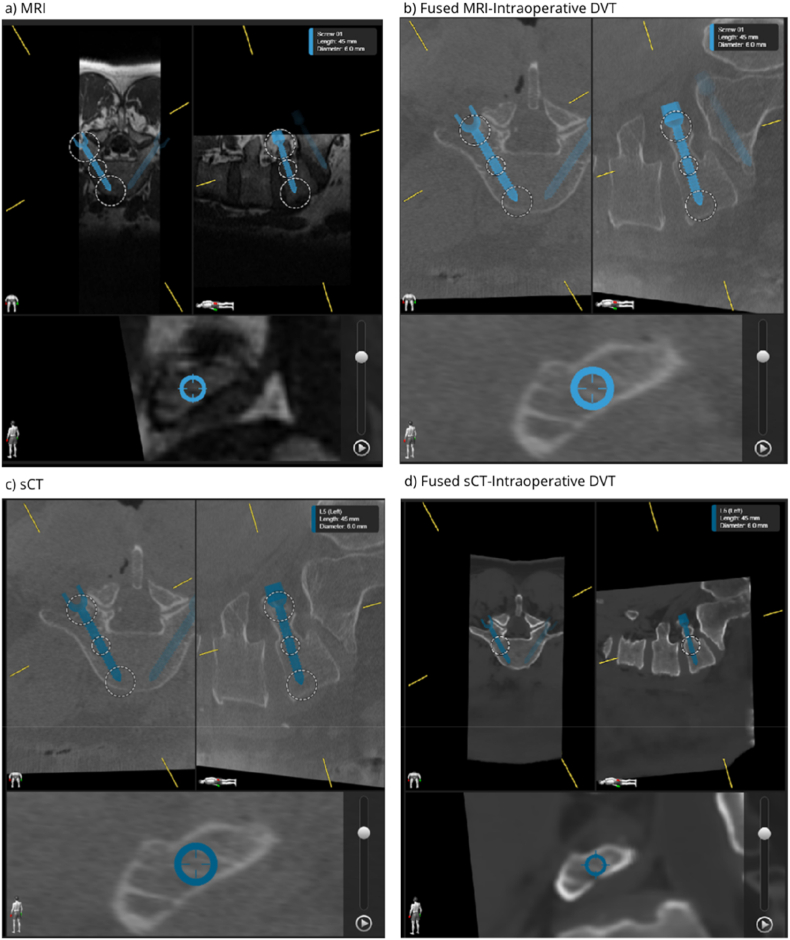
Example images for the different image modalities showing the transverse, sagittal, and trajectory view along the axis of a planned for the L5 vertebra in a single patient. a) MRI, b) fused MRI with intraoperative DVT, c) sCT, and d) fused sCT with intraoperative DVT.

Screenshots of the fused data including the planned screws shown in the Spine Planning software were used for qualitive analysis. Each screenshot showed the transverse, sagittal, and trajectory view along the axis of the screw, a set of example screenshots of a planned screw in the different image modalities is shown in Fig. 2.

### 2.5 Qualitative evaluation

Qualitative evaluation of each screw in the fused plan was performed by three surgeons with at least 10 years of experience in spinal surgery, including the surgeon who performed the planning. Assessment was performed on each screw, based on the planning philosophy, with the Gertzbein-Robbins classification (Gertzbein and Robbins, 1990). The questionnaire evaluated each screw in three sections: diameter (optimal, too small, too large), length (optimal, too short, too long), screw positioning (optimal, too cranial, too caudal, too medial, too lateral). The screw positioning section of the questionnaire was divided into screw head and screw tip and, in this question, it was possible to select multiple non-optimal outcomes (eg, tip was too medial and too cranial).

### 2.6 Data analysis

The data was processed with Python using Visual Studio Code (V1.74.3, Microsoft, USA). Overall, summary statistics were generated for the evaluation from each surgeon for the two different fused image modalities (MRI and sCT).

Percentage interrater agreement and Cohen’s kappa scores were calculated between surgeons for each section of the questionnaire: Gertzbein-Robbins classification, screw diameter, screw length, and screw positioning.

#### 2.6.1 Statistical analysis - image modality comparison

A chi-squared test for distributions was used to identify if there is a significant difference in the questionnaire results between the two image modalities (MRI and sCT). As the chi-squared test uses an approximation to derive a p-value, an alternative exact method is recommended in literature (Hazra and Gogtay, 2016). Therefore, if the number of independent proportions in the chi-squared test was less than five, a Fisher’s exact test was performed instead. To enable the use of chi-squared or Fisher’s exact tests, the data was simplified to optimal or non-optimal.

## 3 Results

A total of 100 screws were evaluated by each surgeon and the assessment of Gertzbein-Robbins classification, screw diameter, screw length, and screw positioning was captured.

### 3.1 Questionnaire results

#### 3.1.1 Gertzbein-Robbins classification

**Fig. 3:**
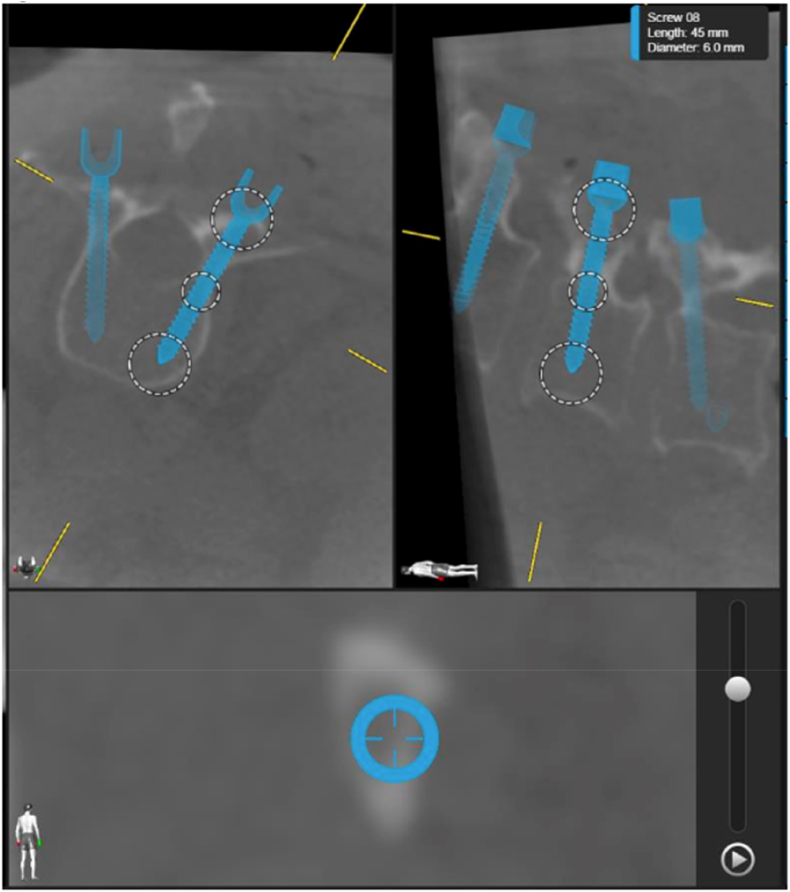
Example image showing in-line screw views for one of the screws rated as C on the Gertzbein-Robbins classification.

The majority of screws were rated as A on the Gertzbein-Robbins classification, where the lowest percentage of A ratings was 85 %. Between 2 and 13 % of screws were classified as B and only two planned screws in the same patient for the MRI image modality received a classification of C by one surgeon. An representative image of a C-rated screw is shown in Fig. 3.

**Fig. 4:**
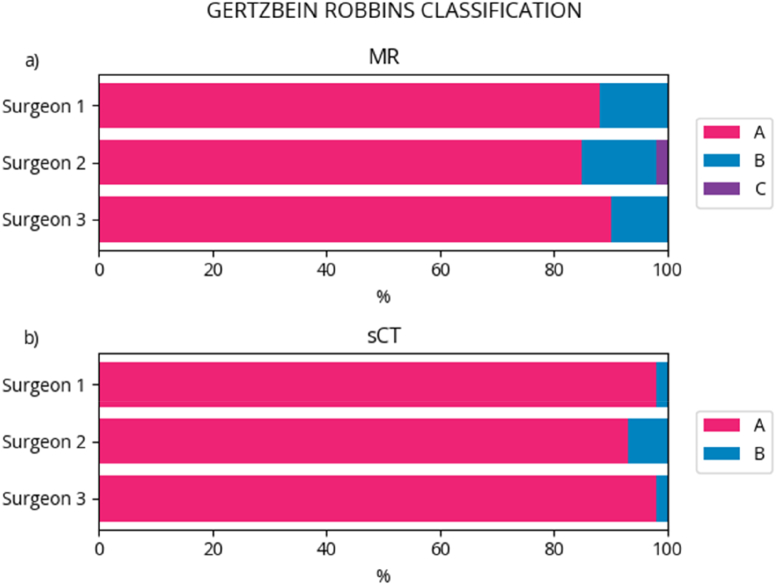
Percentage results of Gertzbein-Robbins classification for the two different image modalities: a) MRI and b) sCT.

In general, the planned screws were given a classification of A more often in the sCT than in the MRI. An interrater agreement between 88 % and 100 % was observed for the screw classification. A summary of the results of Gertzbein-Robbins classification for each screw from the surgeons as well as the percentage interrater agreement for the classification is shown in Table 1. A visual representation of the screw classification is provided in Fig. 4a and b.

**Table 1:** Gertzbein-Robbins Classification for each screw from each surgeon and the percentage interrater agreement.

Gertzbein-Robbins Classification	MRI			sCT		
	Surgeon 1	Surgeon 2	Surgeon 3	Surgeon 1	Surgeon 2	Surgeon 3
A	88	85	90	98	93	98
B	12	13	10	2	7	2
C	0	2	0	0	0	0
D	0	0	0	0	0	0

E	0	0	0	0	0	0

**Interrater agreement**						

Surgeon 1-2	88 %			95 %		
Surgeon 1-3	90 %			100 %		

Surgeon 2-3		93 %			95 %

When separated by vertebra, a slightly lower percentage of A rated screws was observed at L3. The number of screws and mean Gertzbein-Robbins classification as a percentage from the three observers is shown in Table 2.

**Table 2:** Total number of screws per vertebral level with respective mean Gertzbein-Robbins Classification from each observer.

Vertebra	Number of screws	Gertzbein-Robbins Classification: MRI					Gertzbein-Robbins Classification: BoneMRI				
		A	B	C	D	E	A	B	C	D	E
L2	4	83 %	17 %	0 %	0 %	0 %	83 %	17 %	0 %	0 %	0 %
L3	10	77 %	20 %	3 %	0 %	0 %	77 %	20 %	3 %	0 %	0 %
L4	32	89 %	11 %	0 %	0 %	0 %	89 %	11 %	0 %	0 %	0 %
L5	38	85 %	14 %	1 %	0 %	0 %	85 %	14 %	1 %	0 %	0 %
S1	16	100 %	0 %	0 %	0 %	0 %	100 %	0 %	0 %	0 %	0 %

#### 3.1.2 Screw diameter

Between 97 % and 100 % of screw diameters were rated as optimal by the surgeons in both image modalities. Similarly high interrater agreements were observed. Where the diameter was rated non-optimal, no screws were rated as too thin, only too thick. A summary of the results for the screw diameter assessment of each screw from the surgeons and the percentage interrater agreement for the evaluation is shown in Table 3.

**Table 3:** Screw diameter assessment for each screw from each surgeon and the percentage interrater agreement.

Screw Diameter	MRI			sCT		
	Surgeon 1	Surgeon 2	Surgeon 3	Surgeon 1	Surgeon 2	Surgeon 3
Optimal	97	99	98	100	100	99
Too thick	3	1	2	0	0	1

Too Thin	0	0	0	0	0	0

**Interrater agreement**						

Surgeon 1-2	96 %			100 %		
Surgeon 1-3	97 %			99 %		

Surgeon 2-3		98 %			99 %	

#### 3.1.3 Screw length

Screw length was rated as optimal in at least 92 % of the planned screws with between 1 % and 7 % more screws being rated as optimal for the MRI image modality. The majority of non-optimal screws were too short, with only two surgeons rating one screw as too long. Across the screw length data, the interrater agreement was very high, between 94 % and 98 %. A summary of the results for screw length for each screw from the surgeons and the percentage interrater agreement for the evaluation is shown in Table 4.

**Table 4:** Screw length assessment for each screw from each surgeon and the percentage interrater agreement.

Screw length	MRI			sCT		
	Surgeon 1	Surgeon 2	Surgeon 3	Surgeon 1	Surgeon 2	Surgeon 3
Optimal	94	99	95	94	92	94
Too short	5	1	4	6	8	6

Too long	1	0	1	0	0	0

**Interrater agreement**						

Surgeon 1-2	95 %			96 %		
Surgeon 1-3	95 %			98 %		

Surgeon 2-3		94 %			98 %	

#### 3.1.4 Screw positioning

The screw positioning was rated as optimal for MRI planned screws between 74 % and 78 % of screws. Alternatively for the sCT, at least 93 % of the planned screws were rated as optimal. The lowest interrater agreement percentage identified was 83 %. A summary of the results showing the positioning ratings as optimal or non-optimal with the interrater agreement is shown in Table 5.

**Table 5:** Optimal and non-optimal screw positioning results for each screw from each surgeon and the percentage interrater agreement.

Screw positioning	MRI			sCT		
	Surgeon 1	Surgeon 2	Surgeon 3	Surgeon 1	Surgeon 2	Surgeon 3
Optimal	77	74	78	98	97	93
Non-Optimal	23	26	22	2	3	7

**Interrater agreement**						

Surgeon 1-2	83 %			97 %		
Surgeon 1-3	85 %			93 %		

Surgeon 2-3		86 %			92 %	

The most frequently selected reason for a non-optimal screw positioning was “tip too caudal” in the MRI image modality, which was selected 19 times. A summary table showing how often the selected reasons for non-optimal screw positioning for the MRI and sCT were chosen is provided in the supplementary information of this article.

### 3.2 Interrater agreement – Cohen’s kappa

For all questionnaire sections, Cohen’s Kappa was calculated and are shown in Table 6.

**Table 6:** Cohen's kappa scores for different variables assessed.

Variable compared	Image modality	Surgeon 1 - Surgeon 2	Surgeon 1 - Surgeon 3	Surgeon 2 - Surgeon 3
G-R Classification	BoneMRI	0,43	1,00	0,43
	MRI	0,49	0,49	0,68
Diameter	BoneMRI	1,00	0,00	0,00
	MRI	−0,02	0,39	0,66
Length	BoneMRI	0,69	0,82	0,79
	MRI	0,19	0,35	−0,02
Position	BoneMRI	0,52	0,59	0,48
	MRI	0,53	0,57	0,40

It was identified that a large portion of the data suffered from the “Kappa paradox.” The Kappa paradox is a limitation to the Cohen’s kappa calculation where, due to the distribution of the agreement, a high percentage interrater agreement results in an invalid low Cohen’s Kappa score (Feinstein and Cicchetti, 1990; Dettori and Norvell, 2020).

### 3.3 Statistical analysis - image modality comparison

A statistical significance between the image modalities was identified in the Gertzbein-Robbins classification (for surgeons 1 and 3), the screw length (for surgeon 2) and screw positioning (all surgeons). As the sCT generally showed better results in the survey than the MRI-only technology (see Tables above), the significant difference here suggests that sCT has some advantage in the assessment of screw positioning. A summary table that details which test was used and the p-value is shown in Table 7**.**

**Table 7:** Statistical test applied and p-values for the different sections of the questionnaire for each surgeon, statistically significant results are highlighted in blue.

Questionnaire section	Surgeon 1		Surgeon 2		Surgeon 3	
	Test	P-value	Test	P-value	Test	P-value
G-R Classification	Fisher's	0,01	Chi	0,07	Fisher's	0,03
Screw Diameter	Fisher's	0,25	Fisher's	1,00	Fisher's	1,00
Screw Length	Chi	1,00	Fisher's	0,03	Chi	0,76
Screw Position	Fisher's	<0.001	Chi	<0.001	Chi	<0.001

## 4 Discussion

CT imaging is currently considered the gold standard for assessment of bony structures for screw planning (Omar et al., 2020; Sarwahi et al., 2016); however, it increases the radiation exposure of the patient. The current study qualitatively evaluated lumbar screw planning using novel MRI-based technologies. Three surgeons assessed 200 pedicle screws planned in the sacral lumbar region across two different MRI technologies (100 screws/modality): a novel isotropic MRI sequence and an sCT generated from a T1w-MGE MRI sequence. Each planned screw was reviewed using a simple questionnaire which captured the Gertzbein-Robbins score and the qualitive assessment of the reviewer for screw diameter, screw length, and screw positioning.

The Gertzbein-Robbins classification demonstrated that the image modalities were excellent for planning of pedicle screws, where all but two screws were classified as clinically acceptable (classified as A or B (Vardiman et al., 2020)) with a high interrater agreement. In general for all surgeons, a rating of A was identified more often in the sCT planned screws than the MRI planned screws. At the L3 vertebra, fewer screws were given a rating of A. The reduction in pedicle diameter in the more cranial vertebra may be the cause of this result; however, due to the small number of screws in the upper lumbar region (4 screws in L2, and 10 screws in L3), the results are not conclusive and further investigation is required. In results for two of the three surgeons, a significant difference was identified in the results for Gertzbein-Robbins classification between the two image modalities. These results demonstrate that it is possible to use either technology to plan pedicle screws and in some cases there may be some added benefit to using sCT.

The results for optimal size of the diameter and length were high, where at most 8 % of screws were rated as non-optimal. Naturally, as the results were optimal for so many cases, the interrater agreement was also very high (at least 94 %). The consistency in the results for screw diameter may partially be explained by the training and the preferences of the surgeons. Therefore, there is some limitation on using diameter as a measure of the quality of the screw. In contrast, screw length is dependent on the visualization of the anatomy and screw planning at the anterior of the vertebral body. Generally for diameter and length, there was little difference between the two image modalities. There was only one case where a significant difference was identified between the modalities, in screw length for a single surgeon. As shown in Table 3, six screws were rated as too thick in the MRI, whereas only one screw was rated as too thick in sCT. This suggests that in sCT, the cortical bone can be detected more easily, which may lead to a lower rate of pedicle perforation. Given the results in Table 4, the planning of screw length may be slightly easier when using sCT compared to MRI; however, from this study alone it is not clear why in sCT more screws are rated as too short. Overall, the generally high optimal results indicate that planning of screw diameter and screw length is feasible when using either MRI and sCT.

For screws planned using MRI, the screw positioning was rated as optimal in approximately 75 % of cases with interrater agreement at or above 83 %. Alternatively, the sCT modality showed a higher rate of optimal positioning, 92 % and above, with an interrater agreement between 92 % and 97 %. Although optimal results for screw positioning are lower than the other results in the survey, it is important to note that the magnitude of non-optimal placement was not considered. Therefore, a non-optimal screw may be considered safe but the rating surgeon would prefer to make minor adjustments. This makes the screw positioning assessment a more subjective measure compare to the Gertzbein-Robbins classification, the screw diameter, and the screw length. A statistically significant difference was seen between the two modalities in the results of screw positioning for all three surgeons. This would imply that screw positioning is optimal often in screws planned using the sCT compared to the screws planned using MRI. The most common reason in the MRI modality for non-optimal placement was “tip too caudal” and second most common was “head too caudal.” This is likely the result of reduced visibility during planning of cortical bone, resulting in a more conservatively planned screw (ie,more caudally into the vertebral body). Although there appears to be more difficulty in planning on MRI, the overall results still show good results and that the majority (75 %) of screws can be planned with optimal positioning.

One limitation of this study is that there was no comparison to the current clinical gold standard, CT imaging. Although no direct comparison can be made, the sCT technology used in this article has previously demonstrated a good comparison to CT in the lumbar spine (Davidar et al., 2023; Morbée et al., 2021) and other spinal regions (Florkow et al., 2022; van der et al., 2022). The differences between MRI and CT for screw planning has been previously assessed by measuring pedicle width and length (Omar et al., 2020). In this study, pedicle widths and lengths were measured and showed a significant difference between the two modalities. An important contrast between this study (Omar et al., 2020) and the current study is that the performed study evaluated screw positions rather than just measurements. As screw planning is a multifactorial process, capturing the subjective opinion across multiple surgeons may provide a more holistic and clinically relevant view of the screw planning.

A further limitation to this study is that it was a limited preliminary study with only 22 patients enrolled where a single surgeon completed all planning. As this study focused only on the planning stage of surgery, no surgical outcome has been included in this work. In addition, the study did not rate the quality of the MRI and sCT used for planning. Finding out parameters for detection of poor quality of MRI and sCT may identify scans in advance with non-optimal requirements for planning screws and therefore, identify where a preoperative CT is still needed. Finally, within this study an intraoperative CT was acquired, which results in radiation exposure. However, the results of this paper open the door to use MRI and sCT in combination with other intraoperative methods, such as fluoroscopy or registration-based methods, which may further reduce or even completely eliminate the need for ionizing radiation.

## 5 Conclusion

This study has demonstrated that it is possible to reduce the need for a preoperative CT scan for screw planning by using modern MRI based technologies. In doing so, the overall workflow time and cost can be reduced as well as reducing the ionizing radiation exposure for a patient. In many cases, use of modern sequences in MRI scans can successfully enable planning of lumbar pedicle screws. Where the MRI scan alone is not enough for total confidence, sCT can used to supplement the scan and enable effective planning in approximately 9 out of 10 patients. This new technology is a helpful tool for preparing a surgery, but an experienced surgeon is still needed for detecting the non-optimal planned screws during surgery to avoid a serious complication of poor screw positioning.

## Declaration of competing interest

No funding was received for conducting this study. A. Dixon and S. Kling are employees of Brainlab AG, Olof-Palme-Straße 9, 81829 Munich, Germany. P. Seevinck is Founder and CEO of MRIguidance BV, Utrecht, the Netherlands. The other authors have no competing interests.

## Data Availability

The datasets generated during and/or analysed during the current study are available from the corresponding author on reasonable request.
